# Supplementary material for: Continuous bubble streams for controlling marine biofouling on static artificial structures
Source: PeerJ. 2021 Apr 30;9:e11323. doi: 10.7717/peerj.11323 (PMC8092111; doi:10.7717/peerj.11323)
Supplement: Supplemental Information 1 [file peerj-09-11323-s001.docx]

**S1 SHEAR STRESS STUDY: APPROACH & FINDINGS**

**S1.1 Approach**

Shear stress created by the bubble streams was estimated under various combinations of flow rates (low, medium and high) and surface types used in the scouring trials described in this paper. The approach taken here required the determination of fluid velocity profiles relative to plate surfaces (Crimaldi et al. 2002; Koehl 2007). Video footage of bubble streams moving across plate surfaces was obtained to measure bubble rise velocities away from the plate wall; similar approaches have been used to calculate mean free stream velocities in larval settling studies (Crimaldi et al. 2002). Foul release (FR-IS1100) and acrylic (ACR) coated plates (200 x 150 x 20 mm L x W x H) were mounted to high clarity glass sheets creating a cross sectional plane of bubbles 100-200 mm away from the camera lens. The glass sheet and mounted plate apparatus was placed at a height of 100 mm above the diffuser and the plate set at an angle of 22° relative to the horizontal plane. All video footage was taken at 1080p and 240 fps using underwater lights to illuminate flow patterns.

After video footage was obtained for each material and plate orientation, 10 sequential image frames were selected from each trial for analysis. Each image sequence was analyzed in ImageJ and MATLAB to obtain velocity vectors of bubbles in the frontal cross-sectional plane near the plate surface. Bubbles close to the plate surface were assumed to be in the plane of the glass plate and the change in distance of the bubbles along the plate was marked between each image sequence. The change in distance was then divided by the inverse of the frame rate (240 fps) to yield a set of velocity vectors.

Two methods were used to estimate shear stress depending on the distance from panel surface (Table S1): The Law of the Wall equation (Menesses et al. 2017; Massel 1999) and Fundamental Viscosity equation (Munson et al. 2013). Shear stress values associated with different flow rates (low, medium and high) was calculated using velocity profile data, the Law of the Wall equation and fundamental fluid viscosity equation. The fundamental fluid viscosity equation was used when the bubbles were considered to be in contact with the wall surface (y < 1mm), where the Law of Wall equation isn’t valid (Menesses et al. 2017; Munson et al. 2013). Bubble velocities were relatively constant along the plate surface, assuming 1-dimensional flow allowing the velocities in planes parallel to the plate surface to be averaged. To calculate the velocity of the bubbles away from the wall (along y-axis perpendicular to plate surface) the same approach was used. Figure S1 shows a sample image used for calculating the velocity profiles. Previous studies have used a similar approach for calculating the fluid velocity profile, however, it should be highlighted that these studies used PIV techniques and image processing programs to more precisely compute the velocity vectors (Menesses et al. 2017, Liu et al. 2016). We therefore advise caution regarding the accuracy of our estimates and they should be treated as approximates only. Additionally, our calculations are only applicable to highly simplified fluid flow scenarios; the details of the underlying assumptions used to make these simplifications can be found in Munson et al. (2013) and Massel (1999).

*Table S1-1. Equations used for shear stress calculations. Here ‘y’ denotes the distance from the plate surface. See Menesses et al. (2017) and Munson et al. (2013) for the equation definitions.*

| **Method** | **Comparison** | **Equation** |
| --- | --- | --- |
| Law of the Wall | Flow vs. Shear Stress (y > 1 mm) | $\bar{u}_{x}K\sqrt{\frac{\rho}{\tau}}=ln(\frac{y}{y_{c}})$ |
| Fundamental Viscosity | Flow vs. Shear Stress (y < 1 mm) | τ = μ$\frac{\Delta u_{x}}{\Delta y}$ |


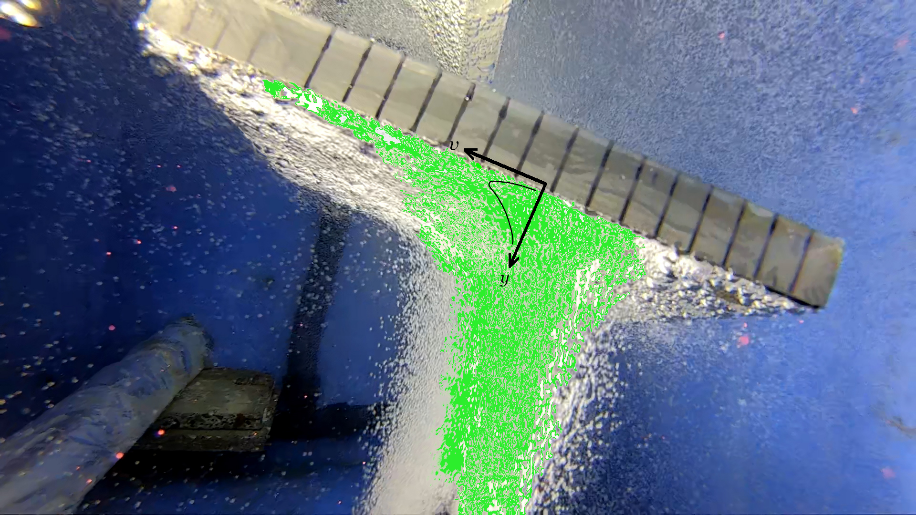


*Figure S1-1: Bubble velocity profile image with coated plate at an angle of 22°. Green contour represents bubbles in the frontal plane used to calculate velocities.*

**S1.2 Findings**

The shear stress values calculated for all experimental trials ranged between 176 ± 71 mPa at low flow rates to 415 ± 97 mPa at high flow rates (Table S2). The higher flow rate bubble trials were excluded from the analysis because of the turbulent behaviour of the flow patterns from the image sequences; bubbles were found to be moving in and out of the glass sheet plane making it impossible to track the bubbles movement along the plates.

*Table S1-2. Shear stress values estimated for bubble streams moving across fouling release (FR) and acrylic (ACR) panels positioned at an angle of 22° and 100mm above diffusers during low (0.9 L hr^-1^ cm^-^²), medium (1.7 L hr^-1^ cm^-^²) and high (2.6 L hr^-1^ cm^-^²) flow rates. Standard deviation (STD) values were calculated using sample STD with a 95% confidence interval. All shear stress values below were calculated using the Law of the Wall Model.*

| **Flow** | **Material** | **Shear stress (mPa)** | **STDev** | **95% CI** |
| --- | --- | --- | --- | --- |
| Low | FR-IS1100 | 305 | 138 | 135 |
| Low | ACR | 176 | 24 | 24 |
| Medium | FR-IS1100 | 385 |  |  |
| Medium | ACR | 345 | 89 | 87 |
| High | ACR | 415 | 99 | 97 |

**S1.2 References**

Crimaldi JP, Thompson JK, Rosman JH, Lowe RJ, Koseff JR. 2002. Hydrodynamics of larval settlement: the influence of turbulent stress events at potential recruitment sites. Limnology and Oceanography 47:1137-1151. doi: [10.4319/lo.2002.47.4.1137](https://www.researchgate.net/deref/http%3A%2F%2Fdx.doi.org%2F10.4319%2Flo.2002.47.4.1137?_sg%5B0%5D=E1-71Re3xtEaBG7aTyHYH8cX_KwcKCQQfI5r9EYjFgyir0wtfkht8tQyIELg_0mykrSbdWABb6GazyDtH5VTMRpIMA.ImRz_rQa8JZXkoI7HnHw2Z8GMDF9jerssQnM_W_GYmVyJ4LFQv5QeQjVK2-M9pPwPfmDeAvLvihOXp2FobsPkQ)

Koehl MRA. 2007. Mini review: Hydrodynamics of larval settlement into fouling communities. Biofouling 23:357-368. [doi:10.1080/08927010701492250](https://doi.org/10.1080/08927010701492250)

Liu L, Yan H, Zhao G, Zhuang J. 2016. Experimental studies on the terminal velocity of air bubbles in water and glycerol aqueous solution. Experimental Thermal and Fluid Science 78:254-265. [doi:10.1016/j.expthermflusci.2016.06.011](https://apc01.safelinks.protection.outlook.com/?url=https%3A%2F%2Fdoi-org.ezproxy.canterbury.ac.nz%2F10.1016%2Fj.expthermflusci.2016.06.011&data=02%7C01%7CGrant.Hopkins%40cawthron.org.nz%7C39d61ce2351c4fd0a7b308d84dff001d%7C0ed55d7825dd4776947a20158de7657d%7C0%7C0%7C637345104911600198&sdata=9z0ra0wzbKfsF%2F5Gn%2BRNXTwuxx%2FsnLhABMJVWx9c%2B8U%3D&reserved=0)

Massel, SR 1999. Fluid mechanics for marine ecologists. Springer, New York; Berlin.

Menesses M, Belden J, Dickenson N, Bird J. 2017. Measuring a critical stress for continuous prevention of marine biofouling accumulation with aeration. Biofouling 33: 703-711. doi:10.1080/08927014.2017.1359574

Munson BR, Okiishi TH, Huebsch WW, Rothmayer AP. 2013. Fundamentals of fluid mechanics, 7th edn, John Wiley & Sons, Inc, Hoboken, NJ.
