# Supplementary material for: Continuous bubble streams for controlling marine biofouling on static artificial structures
Source: PeerJ. 2021 Apr 30;9:e11323. doi: 10.7717/peerj.11323 (PMC8092111; doi:10.7717/peerj.11323)
Supplement: Supplemental Information 2 [file peerj-09-11323-s002.docx]

**S2 Model organisms: spawning and rearing procedures**

**S2.1 *Ciona* *savignyi***

For each trial, approximately 15 reproductively mature *Ciona* were collected from SAS in Nelson Marina (41°15'32.39"S, 173°16'52.74"E) and held in a flow-through aquarium (18°C) for 48-120 hrs. The aquarium was supplied with 10 L seawater h^−1^ via a 1000-L recirculating system held at 18 ± 1 °C and 34 ± 1 psu. The tunicates were fed daily with bulk-cultured microalgae. During this acclimation period, *Ciona* were visually monitored for reproductive condition (the abundance of eggs and sperm are discernible through the tunic) and general health (colour and turgidity). Gravid individuals with densely packed egg and sperm ducts were spawned according to Cirino et al. (2002). After incubating at 18°C for 1 h, fertilized eggs were rinsed (filtered seawater, FSW) on a 20-µm sieve, transferred to a glass Petri dish along with 25 mL FSW, then held at 18°C for 24 h to hatch. Hatched larvae were placed in a conical flask and diluted with FSW to yield desired larval densities of ca. 1 larvae mL^-1^.

**S2.2 *Crassostrea gigas***

Oyster larvae were attained from a commercial oyster hatchery (Cawthron Aquaculture Park). Adult ripe Pacific oysters were strip-spawned and gametes fertilised according to Allen & Bushek (1992). Following 24 h incubation, larvae were stocked at high density (~ 400 mL^-1^) in 170-L cylindro-conical tanks and reared in flow-through conditions. Treated seawater (1 µm filtered, UV-treated seawater maintained at 24°C) previously enriched with hatchery-grown microalgae was continuously supplied to the larval rearing tanks. Developing larvae were periodically graded with nylon screens of increasing size until larvae reached competence for settlement (~ 350 µm shell length), around 17 d post-fertilisation. Larvae were placed on a nylon screen, wrapped in damp paper towels and chilled (around 4°C). Prior to use in trials, larvae were placed in 20 L of 18°C FSW for 30 mins.

Pilot trials were undertaken to identify the most reliable method to facilitate the settlement of *Ciona* and *Crassostrea* larvae on the acrylic and fouling release experimental panels. For *Ciona*, this involved filling a 90 x 15 mm (ca. 95 mL) Petri dish to overflowing with the FSW + larvae solution. Experimental panels where placed facing downwards on the Petri dish, resulting in the centre of the plate coming into direct contact with the larvae solution. Due to the downward swimming behaviour of *Crassostrea* observed by hatchery staff, the petri dish containing larvae and FSW was inverted on top of the experimental panels. For both taxa, larvae were left in contact with panels for 3 h (the 3 h treatment) or for 24 h (for the 120 h treatment). The 3 h panels were treated immediately, while the 120 h panels were left undisturbed for 24 h before being transferred to a 300 L flow-through aquarium for a further 4 days until treatment.

**S2.3 References**

Allen Jr SK, Bushek D. 1992. Large scale production of triploid *Crassostrea virginica* (Gmelin) using ‘stripped’ gametes. Aquaculture 103: 241–251. [doi.org/10.1016/0044-8486(92)90170-P](https://doi.org/10.1016/0044-8486(92)90170-P)

Cirino P, Toscano A, Caramiello D, Macina A, Miraglia V, Monte A. 2002. Laboratory culture of the ascidian *Ciona intestinalis* (L.): a model system for molecular developmental biology research. Marine Models Electronic Record http://comm.archive.mbl.edu/BiologicalBulletin/MMER/cirino/CirBody.html (Accessed on 13 August 2020).
