# Supplementary material for: Continuous bubble streams for controlling marine biofouling on static artificial structures
Source: PeerJ. 2021 Apr 30;9:e11323. doi: 10.7717/peerj.11323 (PMC8092111; doi:10.7717/peerj.11323)
Supplement: Supplemental Information 3 [file peerj-09-11323-s003.docx]

**S3 OUTPUTS FROM STATISTICAL ANALYSES**

*Table S3.1. Results of the generalized linear models for the scouring trial.*

| Factor | Estimate | z.value | p.value |
| --- | --- | --- | --- |
| data: Ciona, acrylic surface, 3 and 120 settelment hours | | | |
| model: ~ Flowrate * Settlementperiodhrs | | | |
| (Intercept) | 2.380 | 8.447 | < 0.001 |
| FlowrateLow | -0.300 | -0.738 | 0.46031 |
| FlowrateMed | -1.099 | -2.483 | 0.01303 |
| FlowrateHigh | -2.380 | -4.080 | < 0.001 |
| Settlementperiodhrs120 | 0.901 | 2.345 | 0.01902 |
| FlowrateLow:Settlementperiodhrs120 | -0.026 | -0.047 | 0.96259 |
| FlowrateMed:Settlementperiodhrs120 | 0.962 | 1.665 | 0.09585 |
| FlowrateHigh:Settlementperiodhrs120 | -0.431 | -0.559 | 0.57582 |
| data: Crassostrea, acrylic and FR surfaces, 3 and 120 settelment hours | | | |
| model: ~ Flowrate * Settlementperiodhrs * SurfaceType | | | |
| (Intercept) | 3.578 | 10.870 | < 0.001 |
| FlowrateLow | 0.197 | 0.424 | 0.67125 |
| FlowrateMed | 0.621 | 1.342 | 0.17973 |
| FlowrateHigh | 0.251 | 0.540 | 0.58912 |
| Settlementperiodhrs1201 | 0.297 | 0.641 | 0.52148 |
| SurfaceTypeFR | -1.969 | -3.928 | < 0.001 |
| FlowrateLow:Settlementperiodhrs1201 | -0.235 | -0.359 | 0.71976 |
| FlowrateMed:Settlementperiodhrs1201 | -0.262 | -0.401 | 0.68832 |
| FlowrateHigh:Settlementperiodhrs1201 | -0.055 | -0.085 | 0.93265 |
| FlowrateLow:SurfaceTypeFR | -0.526 | -0.731 | 0.46466 |
| FlowrateMed:SurfaceTypeFR | -1.537 | -2.055 | 0.03991 |
| FlowrateHigh:SurfaceTypeFR | 1.084 | 1.579 | 0.11430 |
| Settlementperiodhrs120:SurfaceTypeFR | 1.434 | 2.038 | 0.04155 |
| FlowrateLow:Settlementperiodhrs120:SurfaceTypeFR | -1.020 | -1.017 | 0.30919 |
| FlowrateMed:Settlementperiodhrs120:SurfaceTypeFR | -2.386 | -2.124 | 0.03369 |
| FlowrateHigh:Settlementperiodhrs120:SurfaceTypeFR | -3.591 | -3.598 | < 0.001 |
| data: Crassostrea, FR surface, 120 settlement hours | | | |
| model: ~ Flowrate | | | |
| (Intercept) | 3.341 | 6.113 | < 0.001 |
| FlowrateLow | -1.583 | -2.106 | 0.03520 |
| FlowrateMed | -3.564 | -4.034 | < 0.001 |
| FlowrateHigh | -2.311 | -2.979 | 0.00289 |

*Table S3.2. Results of the generalized linear models for the disruption trial.*

| Factor | Estimate | z.value | p.value |
| --- | --- | --- | --- |
| species: Ciona | | | |
| model: ~ Flowrate * SurfaceType | | | |
| FlowrateMed | -21.303 | -0.002 | 0.99838 |
| FlowrateNil | 5.852 | 18.767 | < 0.001 |
| SurfaceTypeFR | 0.000 | 0.000 | 1.00000 |
| FlowrateNil:SurfaceTypeFR | -2.264 | 0.000 | 0.99988 |
| model: ~ Flowrate | | | |
| FlowrateMed | -21.303 | -0.002 | 0.99838 |
| FlowrateNil | 3.588 | 11.665 | < 0.001 < 0.001 |
| species: Crassostrea | | | |
| model: ~ Flowrate * SurfaceType | | | |
| FlowrateMed | 2.037 | 9.702 | < 0.001 < 0.001 |
| FlowrateNil | 5.953 | 39.454 | < 0.001 < 0.001 |
| SurfaceTypeFR | -2.442 | -4.342 | < 0.001 |
| FlowrateNil:SurfaceTypeFR | 2.422 | 4.026 | < 0.001 |
| model: ~ Flowrate | | | |
| FlowrateMed | -0.405 | -0.804 | 0.42160 |
| FlowrateNil | 5.933 | 83.961 | < 0.001 < 0.001 |

*Table S3.3. Results of the statistical models for the vertical surfaces field trials.*

| Factor | Estimate | z.value | p.value |
| --- | --- | --- | --- |
| **model: LOF ~ Surface * Treatment + (1 \| Week)** | | | |
| SurfaceFR-IS1100 | -44.205 | -38,986.22 | < 0.001 |
| TreatmentControl | 91.241 | 80,328.14 | < 0.001 |
| SurfaceFR-IS1100:TreatmentControl | -1.168 | -923.81 | < 0.001 |
| **Factor** | **Estimate** | **z.value** | **p.value** |
| **model: percent macrofouling cover ~ Surface * Treatment** | | | |
| SurfaceFR-IS1100 | -44.205 | -38,986.22 | < 0.001 |
| TreatmentControl | 91.241 | 80,328.14 | < 0.001 |
| SurfaceFR-IS1100:TreatmentControl | -1.168 | -923.81 | < 0.001 |
| **Factor** | **Estimate** | **Std.Error** | **p.value** |
| **model: dry weight ~ Surface * Treatment** | | | |
| (Intercept) | 0.336 | 0.058 | 5.82 |
| SurfaceFR-IS1100 | 3.746 | 0.499 | 7.503 |
| TreatmentControl | -0.272 | 0.058 | < 0.001 |
| SurfaceFR-IS1100:TreatmentControl | -2.434 | 0.527 | < 0.001 |

*Table S3.4. Results of the generalized linear models for the horizontal and angled surfaces field trials.*

| Factor | Estimate | z.value | p.value |
| --- | --- | --- | --- |
| percent: Bare space | | | |
| model: Bare space ~ Surface * Angle + Round | | | |
| (Intercept) | -20.273 | -0.015 | 0.98768 |
| SurfaceIS1000 | 19.817 | 0.015 | 0.98796 |
| SurfaceIS1100 | 20.059 | 0.015 | 0.98781 |
| SurfacePOLY | 20.088 | 0.015 | 0.98780 |
| Angle22° | 19.768 | 0.015 | 0.98799 |
| RoundR2 | -2.143 | -12.905 | < 0.001 < 0.001 |
| RoundR3 | -1.420 | -10.147 | < 0.001 < 0.001 |
| SurfaceIS1000:Angle22° | -19.311 | -0.015 | 0.98827 |
| SurfaceIS1100:Angle22° | -20.661 | -0.016 | 0.98745 |
| SurfacePOLY:Angle22° | -19.370 | -0.015 | 0.98823 |
| percent: Biofilm | | | |
| model: Biofilm ~ Surface * Angle + Round | | | |
| (Intercept) | 3.276 | 7.200 | < 0.001 |
| SurfaceIS1000 | -2.812 | -5.863 | < 0.001 |
| SurfaceIS1100 | -3.088 | -6.082 | < 0.001 |
| SurfacePOLY | -3.205 | -6.731 | < 0.001 |
| Angle22° | -2.883 | -6.019 | < 0.001 |
| RoundR2 | 1.903 | 12.594 | < 0.001 |
| RoundR3 | 1.548 | 11.068 | < 0.001 |
| SurfaceIS1000:Angle22° | 2.449 | 4.689 | < 0.001 |
| SurfaceIS1100:Angle22° | 3.707 | 6.607 | < 0.001 |
| SurfacePOLY:Angle22° | 2.311 | 4.479 | < 0.001 |
| percent: Macrofouling | | | |
| model: Macrofouling ~ Surface * Angle + Round | | | |
| (Intercept)2 | -3.451 | -7.814 | < 0.001 |
| SurfaceIS1000 | -1.118 | -1.360 | 0.17395 |
| SurfaceIS1100 | 0.026 | 0.035 | 0.97205 |
| SurfacePOLY | 0.159 | 0.282 | 0.77831 |
| Angle22° | -0.187 | -0.305 | 0.76023 |
| RoundR2 | -0.094 | -0.297 | 0.76625 |
| RoundR3 | -3.276 | -3.188 | 0.00143 |
| SurfaceIS1000:Angle22° | 0.187 | 0.159 | 0.87363 |
| SurfaceIS1100:Angle22° | -0.546 | -0.525 | 0.59960 |
| SurfacePOLY:Angle22° | 1.188 | 1.553 | 0.12043 |
