# Supplementary material for: Continuous bubble streams for controlling marine biofouling on static artificial structures
Source: PeerJ. 2021 Apr 30;9:e11323. doi: 10.7717/peerj.11323 (PMC8092111; doi:10.7717/peerj.11323)
Supplement: Supplemental Information 4 [file peerj-09-11323-s004.docx]

**S4 UNDERWATER SOUND MEASUREMENTS**

| **0.5 m** | 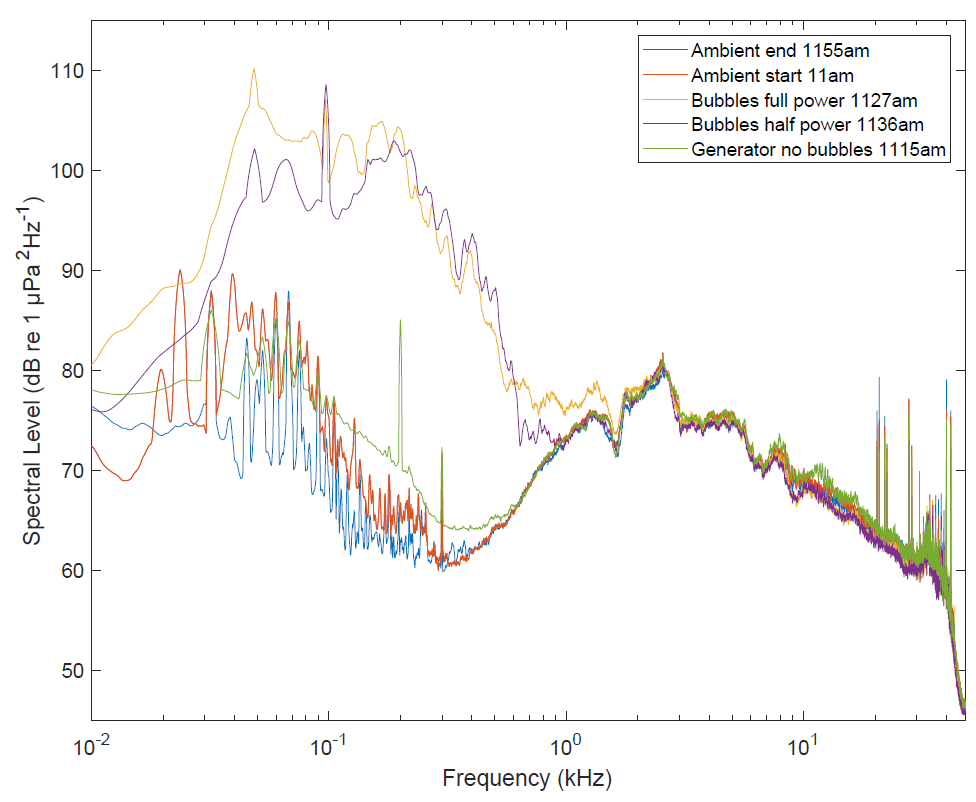 |
| --- | --- |
| **16 m** | 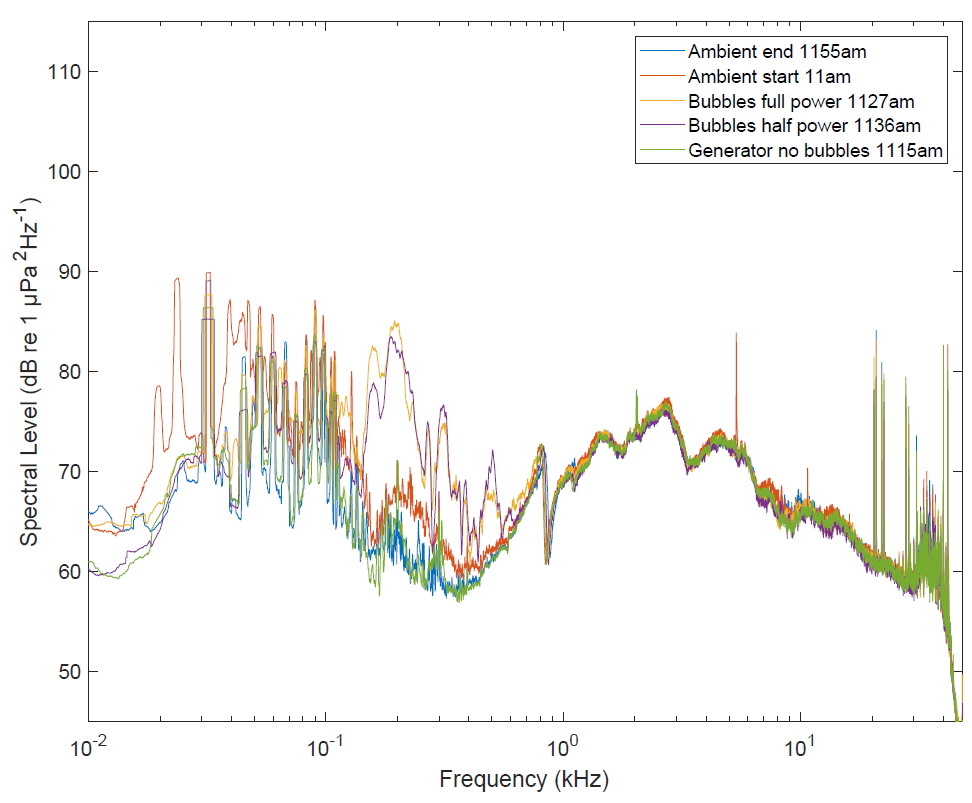 |
| **32 m** | 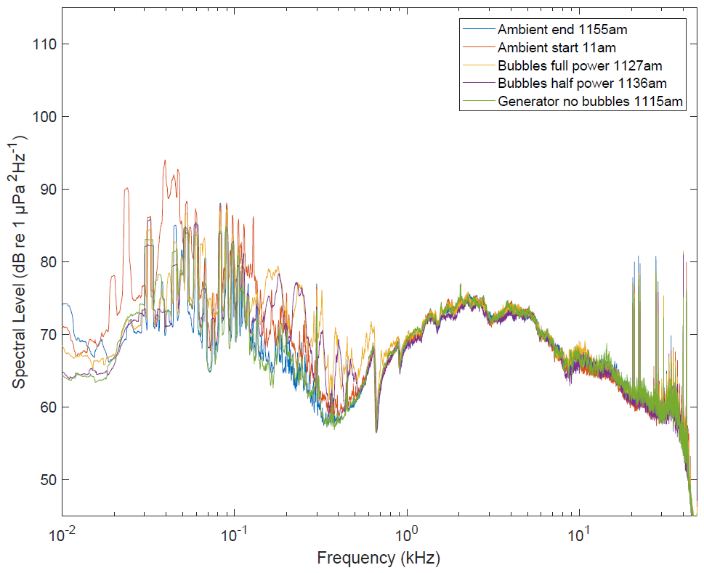 |

*Figure S4.1. Sound field measured approximately 0.5 m (top), 16 m (middle) and 32 m (bottom) from the bubble diffusers. The top panel clearly shows sound levels above ambient; this was less evident at 16 m, and indistinguishable at 32 m from the diffusers. Red arrows indicate where sounds produced from the diffusers can be detected above ambient.*
