# Supplementary material for: Continuous bubble streams for controlling marine biofouling on static artificial structures
Source: PeerJ. 2021 Apr 30;9:e11323. doi: 10.7717/peerj.11323 (PMC8092111; doi:10.7717/peerj.11323)
Supplement: Supplemental Information 5 [file peerj-09-11323-s005.docx]

**S5 Field trial images (horizontal surfaces)**

**S5.1 ROUND 1:** Sampling undertaken on 10 December 2018, following a 46-day deployment. During this deployment, there was a large over settlement of barnacles (species name) on the raft and floats.

|  | CONCRETE | POLYETHYLENE | Intersleek 1000 | Intersleek 1100 |
| --- | --- | --- | --- | --- |
| FLAT | 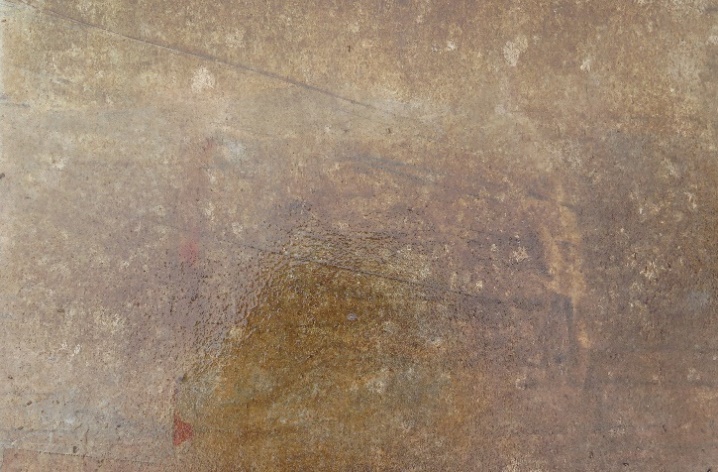 | 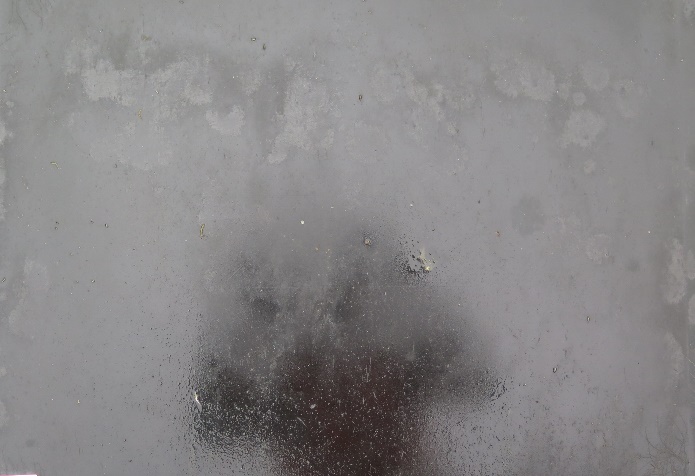 | 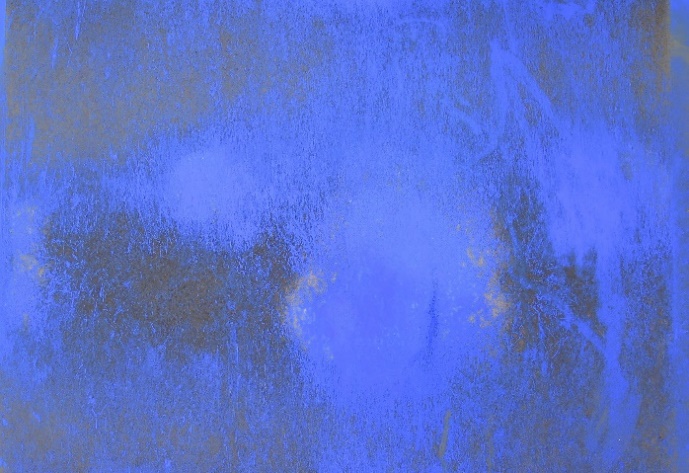 | 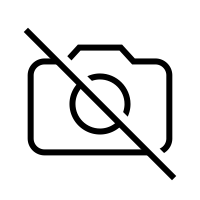 |
|  |  |  |  |  |
| ANGLED | 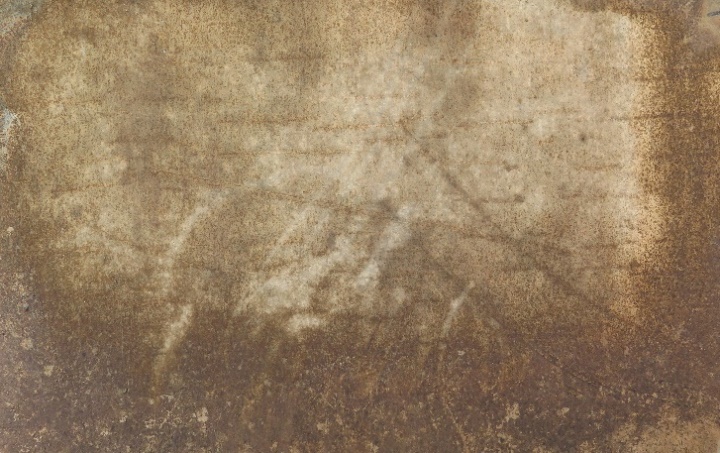 | 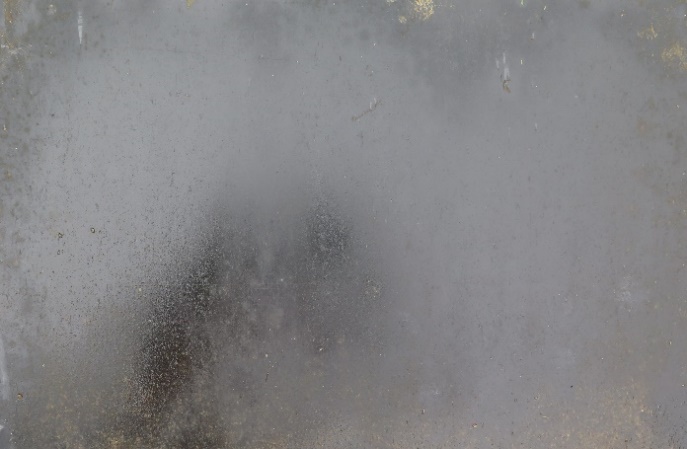 | 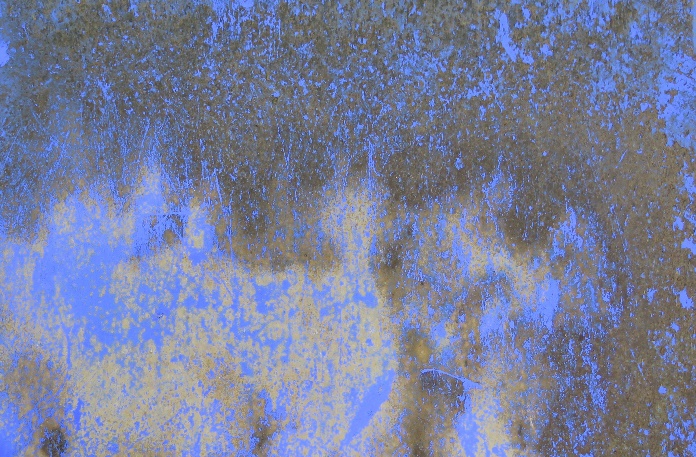 | 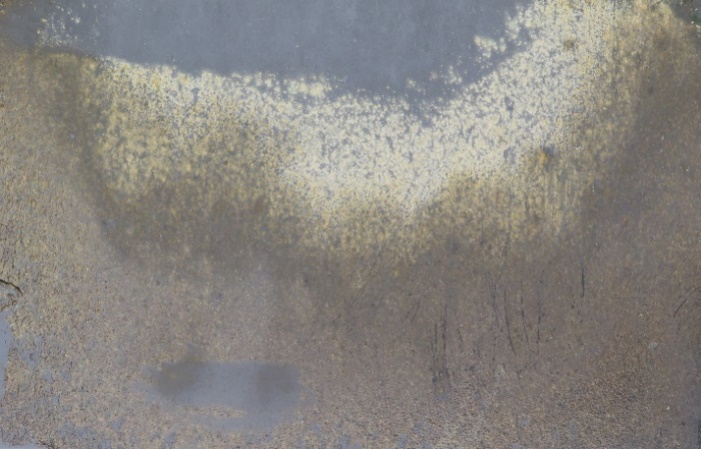 |

**S5.2 ROUND 2:** Sampling undertaken on 27 February 2019, following a 79-day deployment.

|  | CONCRETE | POLYETHYLENE | Intersleek 1000 | Intersleek 1100 |
| --- | --- | --- | --- | --- |
| FLAT | 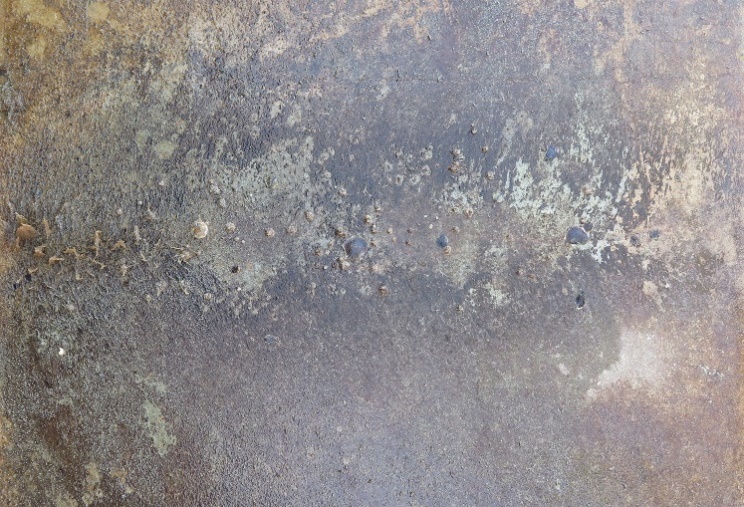 | 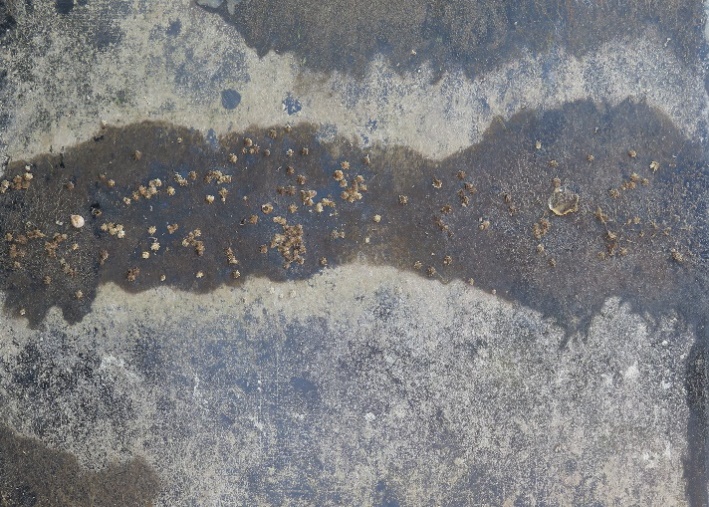 | 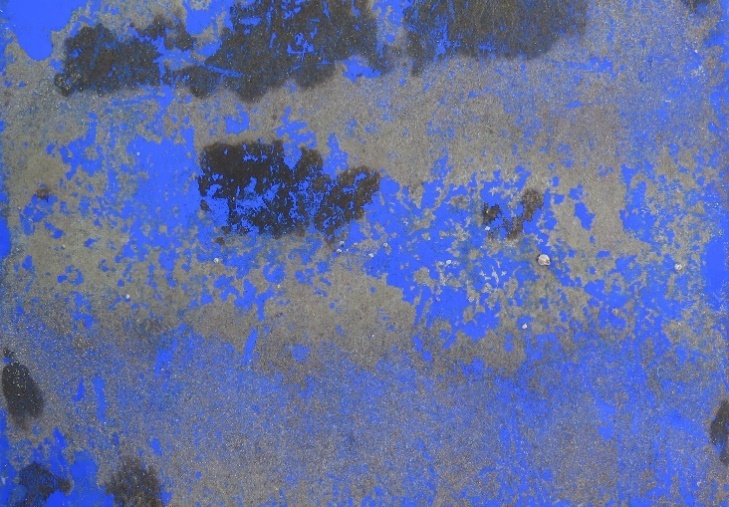 | 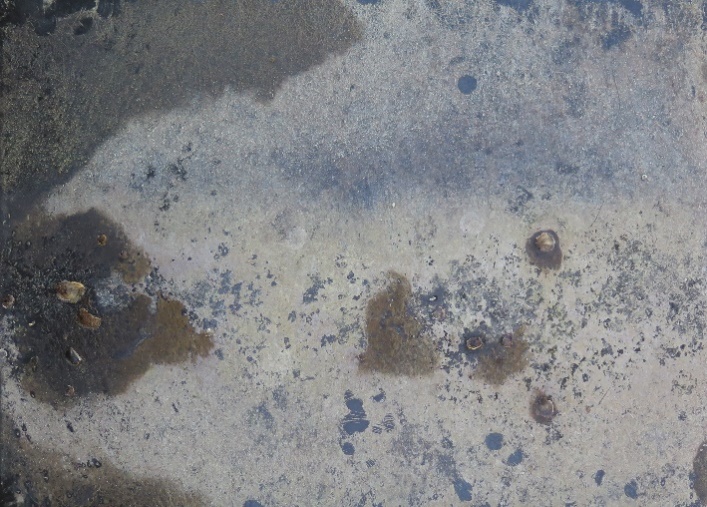 |
|  |  |  |  |  |
| ANGLED | 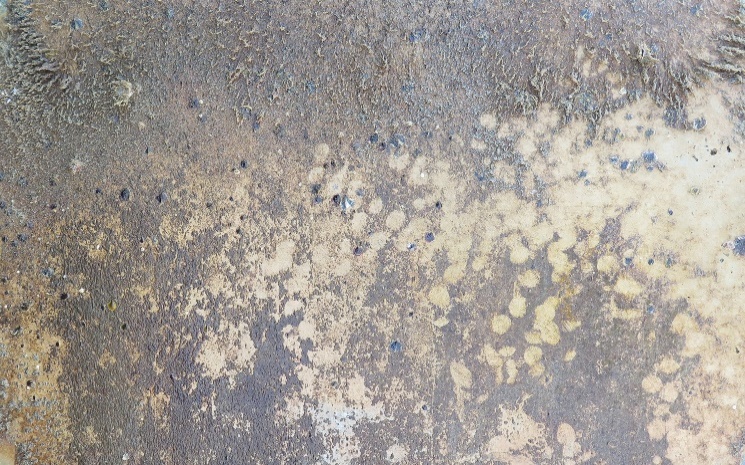 | 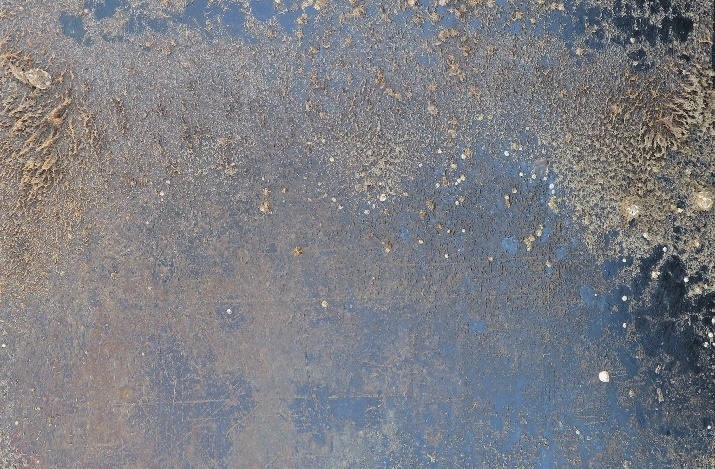 | 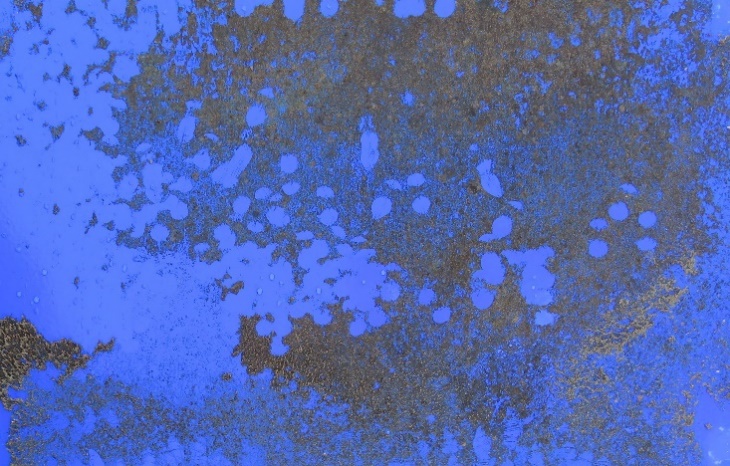 | 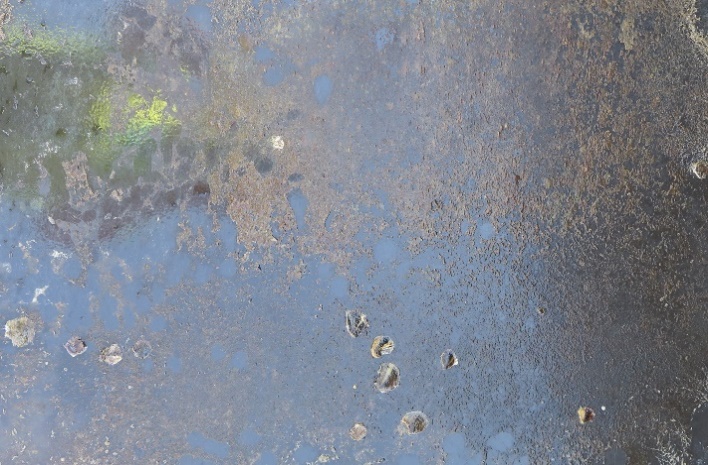 |

**S5.3 ROUND 3:** Sampling undertaken on 22 May 2019, following a 84-day deployment.

|  | CONCRETE | POLYETHYLENE | Intersleek 1000 | | Intersleek 1100 |
| --- | --- | --- | --- | --- | --- |
| FLAT | 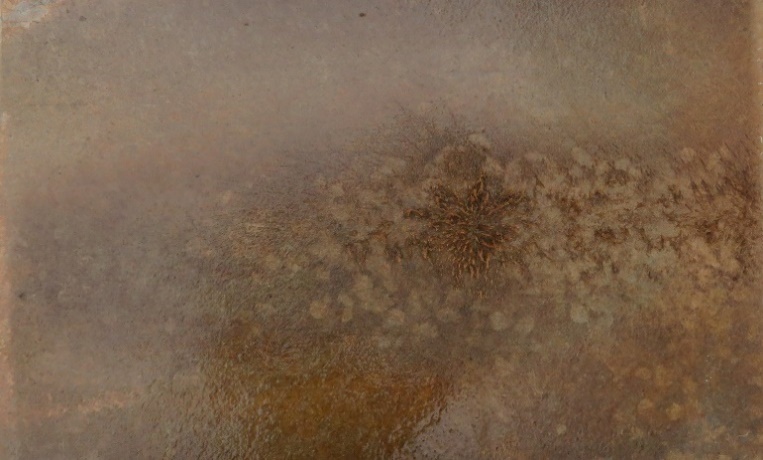 | 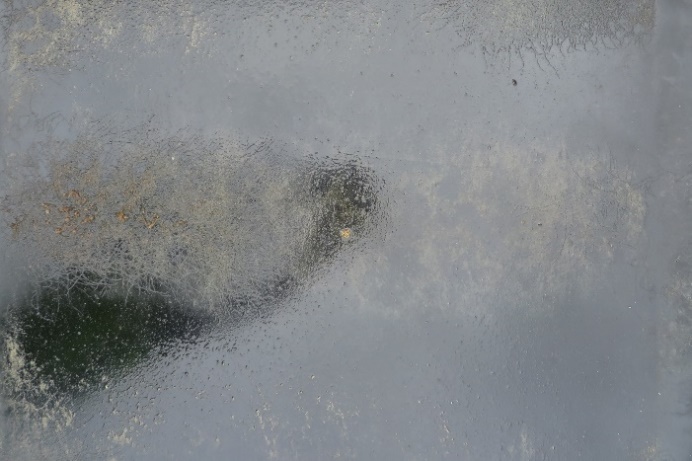 | 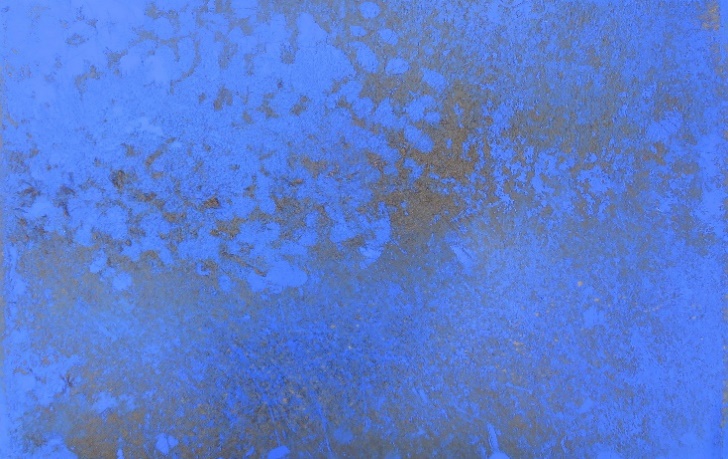 | 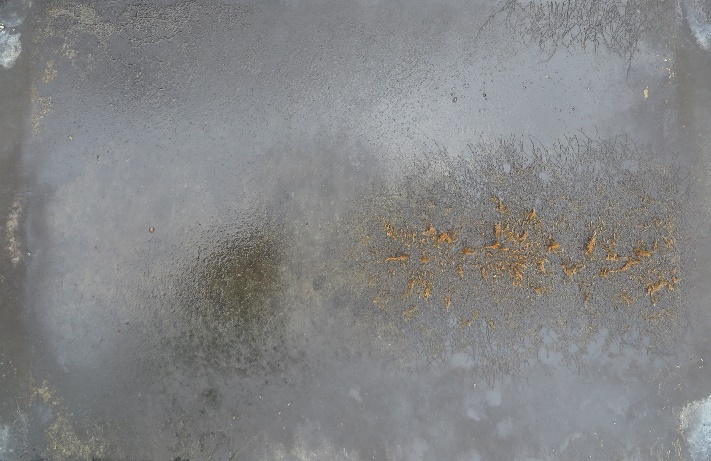 | |
|  |  |  |  |  | |
| ANGLED | 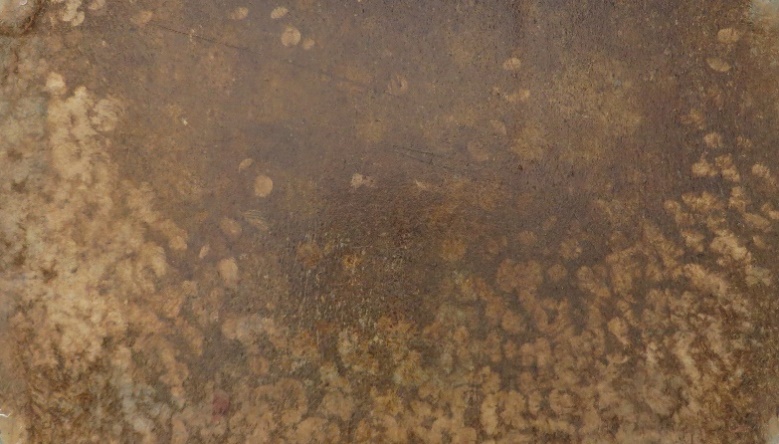 | 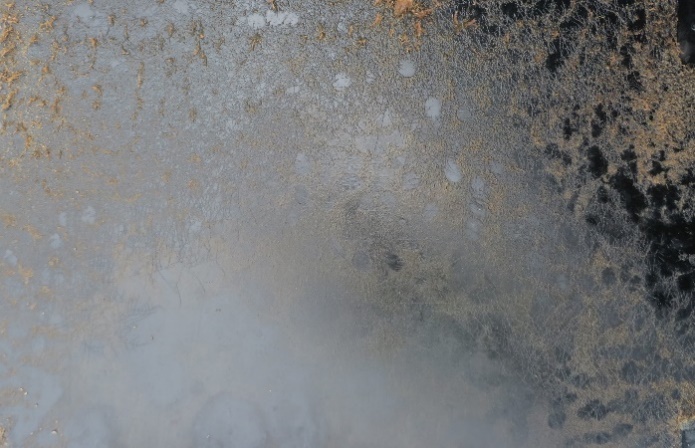 | 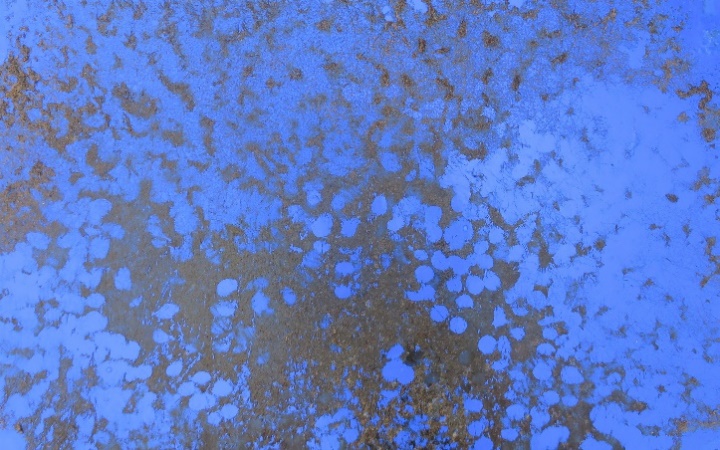 | 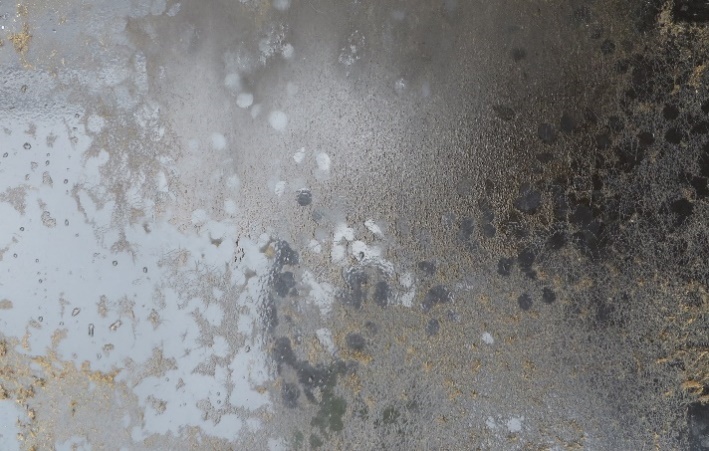 | |

**S5.4 Final round:** Sampling undertaken on 19 September 2019, following a 119-day deployment.

|  | TREATMENT | | CONTROLS | |
| --- | --- | --- | --- | --- |
| POLYETHYLENE | 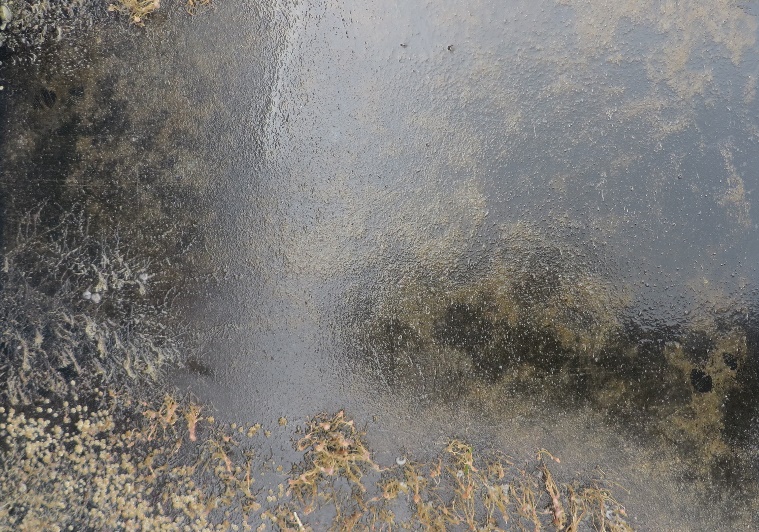 | 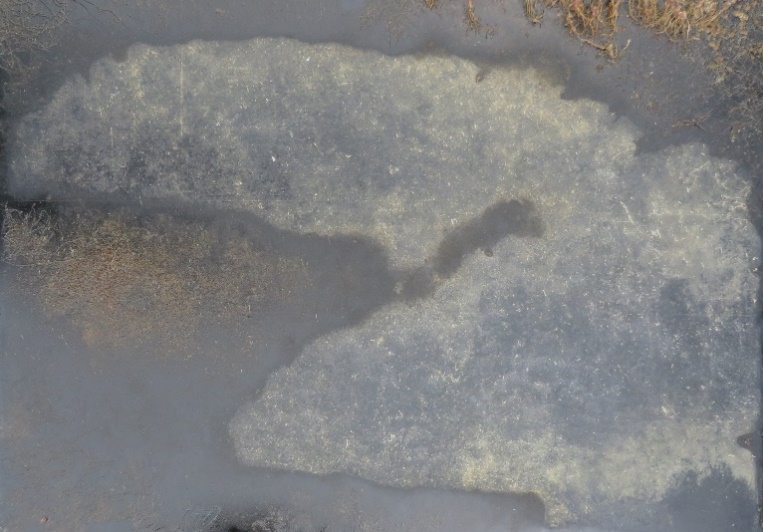 | 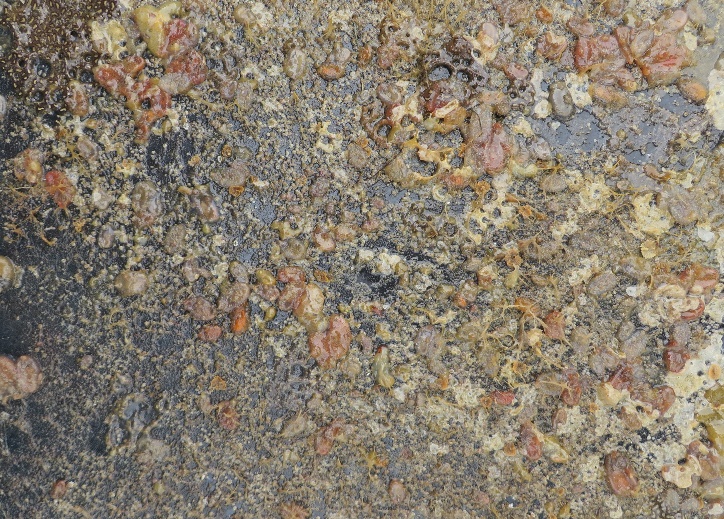 | 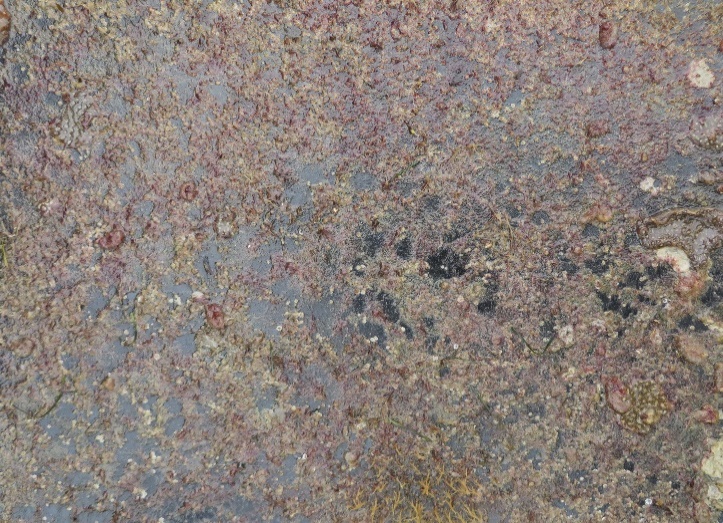 |
|  |  |  |  |  |
| Intersleek 1000 | 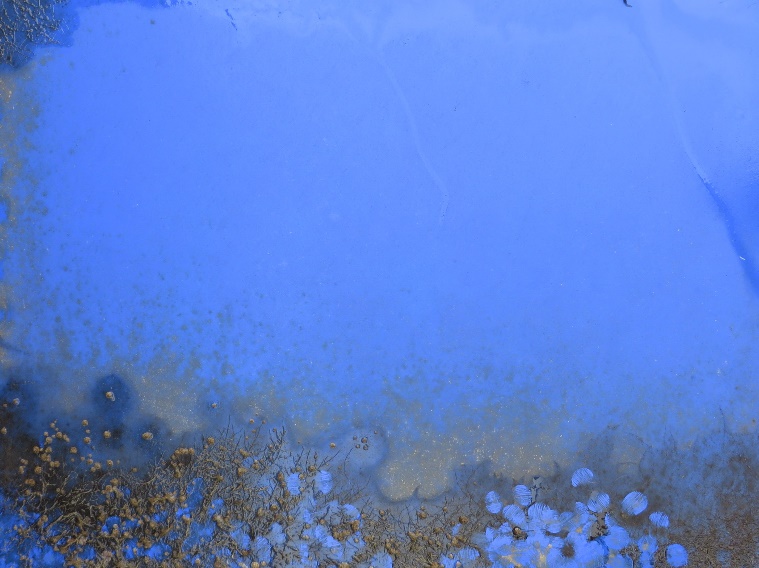 | 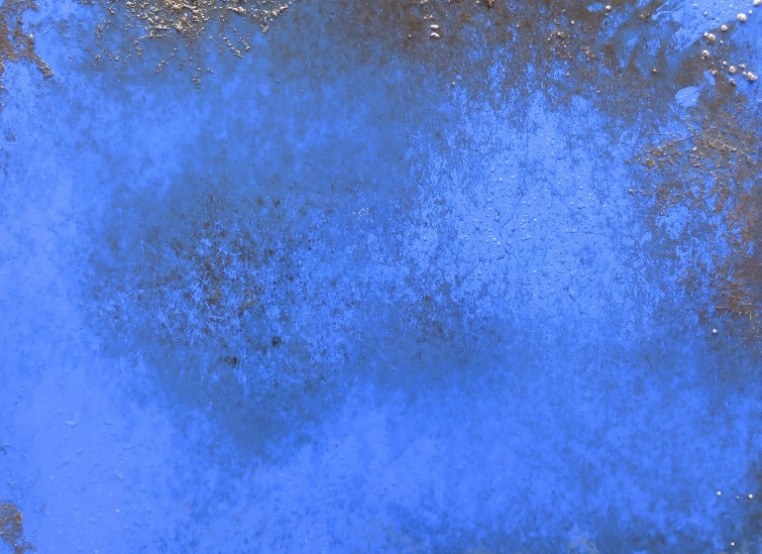 | 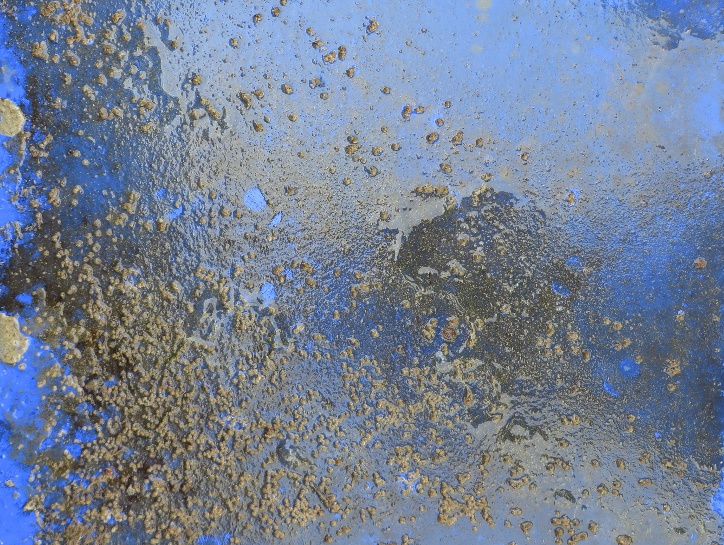 | 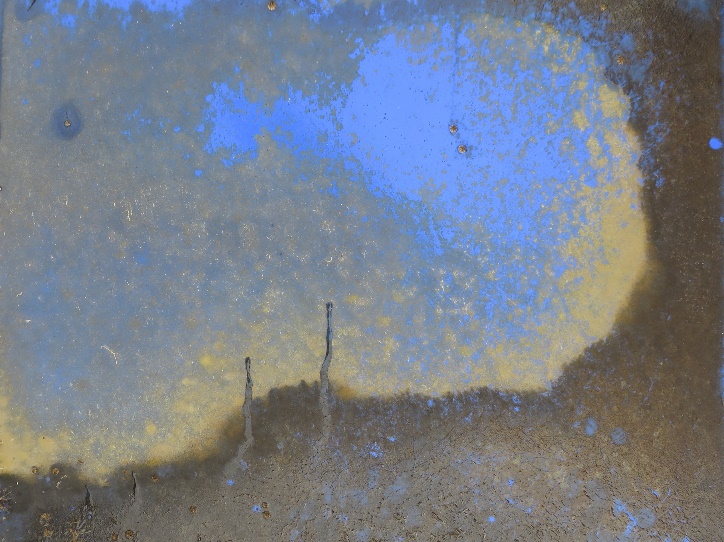 |
